# Note on Crystallization for Alternating Particle Chains

**DOI:** 10.1007/s10955-020-02603-2

**Published:** 2020-07-13

**Authors:** Laurent Bétermin, Hans Knüpfer, Florian Nolte

**Affiliations:** 1 Faculty of Mathematics, University of Vienna, Oskar-Morgenstern-Platz 1, 1090 Vienna, Austria; 2 Institute of Applied Mathematics and IWR, University of Heidelberg, Im Neuenheimer Feld 205, 69120 Heidelberg, Germany

**Keywords:** Crystallization, Ionic crystals, Convexity, Energy minimization, 82B05, 26A51, 74E15

## Abstract

We investigate one-dimensional periodic chains of alternate type of particles interacting through mirror symmetric potentials. The optimality of the equidistant configuration at fixed density—also called crystallization—is shown in various settings. In particular, we prove the crystallization at any scale for neutral and non-neutral systems with inverse power laws interactions, including the three-dimensional Coulomb potential. We also show the minimality of the equidistant configuration at high density for systems involving inverse power laws and repulsion at the origin. Furthermore, we derive a necessary condition for crystallization at high density based on the positivity of the Fourier transform of the interaction potentials sum.

## Introduction

A fundamental question in the theory of crystallization is to understand why many large systems of interacting particles exhibit the spontaneous formation of periodic structures and how they can be explained by energy minimization [10]. Such periodic structures are observed in systems consisting of identical particles but also appear in models composed of different types of particles. For example, ionic compounds exhibit periodic structures, even though different attractive and repulsive interaction potentials between the ions are present [35]. In this paper, we consider prototypical alternating chains of particles where particles of different (resp. same) kind repel (resp. attract) each other at short distance and investigate necessary and sufficient conditions for the optimality of periodic (equidistant) configurations. This consideration is motivated for instance by alternating chains of magnetic domain walls (see e.g. [24]). We note that while one-dimensional model systems do not occur commonly in nature, they can be created by confinement (see e.g. [32]).

In this paper, once the charges are fixed, as well as the interaction between species, we show the optimality of the equidistant configuration at fixed density, among one-dimensional periodic configurations of alternating species in different settings. The novelty of the paper consists in the systematic analysis for the ground state energy of alternating two-particle systems. We assume repulsive interaction at short distances between different species in order to avoid a collapsing of the ground state. We will also show that in the neutral Coulomb case or in the power-law case, the equidistant configuration is the unique minimum of the energy, and we expect this result to hold for a large class of interaction potentials leading to a new kind of *universal optimality* as defined by Cohn and Kumar in [14] for two-component systems (see Conjecture 2.7). We note that the model considered is chosen as a simple prototype model. More general, it would be interesting to derive conditions for periodicity in higher-dimensional systems, to consider systems of more than two different particles or to consider the case of different ratio between the involved species.

For one-dimensional systems of identical particles, Ventevogel and Nijboer [36, 37] have derived several results about the optimality of the equidistant configuration. In their work, interacting potentials are mirror symmetric and correspond to semi-empirical potentials used in molecular simulations (see e.g. [29, p. 624]). In particular, they proved the optimality of the equidistant configuration for convex interaction potentials and Lennard–Jones-type potentials (also called “Mie potentials”) among periodic configurations. A similar result by Radin [19] shows the optimality of an equidistant configuration for the classical (12, 6) Lennard-Jones potential, when the number of points—added alternatively to both sides of the configuration—goes to infinity, and a generalization of this result has been recently shown in [23] where the temperature is added. Another recent result by Bandegi and Shirokoff [4, Sect. 6.1] gives numerical evidences for the global optimality of the equidistant configuration for some values of the density and the parameters of the Morse potential using convex relaxation.

One-dimensional systems involving power-laws and two kind of species have been numerically studied in [25] in a different perspective, changing the species ratio and considering interaction only between species of the same kind. We note that similar studies for binary mixtures or particles have been made in dimension two for dipolar (inverse power law) interaction and the Yukawa potential (see [2] and references therein).

Systems with different types of particles also arise in other models such as e.g. chains of interacting magnetic dipoles (e.g. [5, 33], see also Fig. 2). Also the interaction of stripe type magnetic domains in thin ferromagnetic films can be described in this setting, where the sign of the interaction energy between two interfaces in this model depends on the number of in-between interfaces so that this model can be viewed as a system of alternating particles of two kinds (see [24]). Furthermore, our work can be related to classical models of spin chains, and more precisely to the works of Giuliani et al. on spin lattice models for stripe formation [20, 21]. We note that the type of models investigated in this paper might also be interesting for biological models related to swarming and flocking between different species (although in a dynamical, higher dimensional setting, see e.g. [12, 13, 27]).

Furthermore, one-dimensional quantum models involving nuclei and electrons have also been studied. Brascamp and Lieb [11] as well as Aizenman and Martin [1] gave important results on the optimality of the equidistant configuration concerning the Jellium model, see also [31]. The emergence of crystallized state for one-dimensional system embedded in a periodic energy landscape is furthermore studied in [18] by Friedrich and Stefanelli where it is shown that equidistant ground states are generally not expected. Furthermore, Blanc and Le Bris [9] proved the periodicity of the ground state for the one-dimensional Thomas-Fermi-von-Weizsäcker energy.

For higher dimensional systems, partial progress has been made for special potentials or in restricted settings, especially in two and three dimensions. In the case of only one type of particle, most of the known results in two and three dimensions concern perturbations of hard-sphere potentials or special oscillating potentials (see [8, 10] and references therein). In the special 8- and 24-dimensional cases, crystallization has been shown at fixed density for the class of completely monotone potentials in [15]. In the case of several type of interacting particles, the existing results rely on the design of specific short-range potentials such that the difference of repulsion at short distance forced the particles to be in a certain configuration. This is the main difference between these works and the present paper where the interaction potentials are long-range and standard in molecular simulations. A first proof of crystallization was given by Radin in [30]. Using three different radially symmetric short-range interaction potentials, he proved the minimality of a two-dimensional binary quasiperiodic configuration. Furthermore, Friedrich and Kreutz [16, 17] have shown the optimality of two-dimensional structures with alternating charges. They explored the possibilities to obtain a rock-salt structure or an alternate honeycomb lattice as minimizer of an interaction energy involving only short-range radially symmetric potentials.

## Setting and Statement of Main Results

**Fig. 1:**
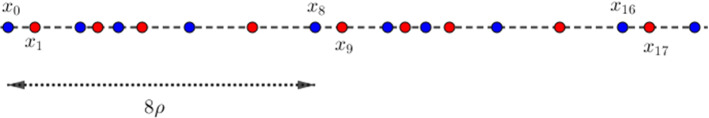
Example of periodic configuration $X\in {\mathcal A}_{8}^\rho$

We consider a one-dimensional chain composed of two different types of particles, located at the positions $x_k \in {\mathbb {R}}$ for $k \in \mathbb {Z}$ (see Fig. 1). Since we are interested in the case when the interaction energy between different types of particles is repulsive at short distance, we will assume that the different particles are ordered alternatively. For technical reasons, we also assume that the particles positions are *N*-periodic for a large even number *N*. We note that our results are independent of *N* which is assumed to be very large. Recall that our goal is to show necessary conditions for minimizers of the considered systems to be 1–periodic.

### Definition 2.1

(*Configurations and energy*) Let $Let N\in 2 \mathbb {N}.$

(i) For $\rho >0$, the class of *N*–periodic configurations with density $\rho$ is denoted by
  $$\begin{aligned} {\mathcal A}_{N}^\rho = \left\{ (x_k)_{k \in \mathbb {Z}}{:} \ {x_0 = 0, x_k < x_{k+1} \text { and } x_{k+N} = x_k +\rho ^{-1} N \quad \forall k \in \mathbb {Z}} \right\} . \end{aligned}$$
  We assume that the particle $x_k$ is of type $\epsilon _k$ where $\epsilon _k :=1$ if $k\in 2\mathbb {Z}+1$ and $\epsilon _k :=2$ if $k \in 2\mathbb {Z}$. The equidistant configuration $e^\rho \in {\mathcal A}_N^\rho$ is denoted by $e^\rho := (k\rho ^{-1})_{k \in \mathbb {Z}}$.
(ii) For $\alpha , \beta \in \{ 1, 2 \}$, let $f_{\alpha \beta } : {\mathbb {R}}\rightarrow {\mathbb {R}}$ be mirror symmetric interaction potentials, i.e $f_{\alpha \beta }(-x)=f_{\alpha \beta }(x)$. The associated energy for ${\mathcal {F}}= (f_{11},f_{22},f_{12})$ is denoted by
  $$\begin{aligned} E_{{\mathcal {F}}}(X):=&\frac{1}{N}\sum _{n=1}^N {\mathop {\mathop {\sum }\limits _{k = -\infty }}\limits _{k\ne n}^{\infty }} f_{\epsilon _n\epsilon _k}(x_{k}-x_n), \qquad \text {for }X = (x_i)_{i \in \mathbb {Z}} \in {\mathcal A}_N^\rho . \end{aligned}$$

We consider configurations that minimize the energy at fixed density. In particular, we say that the equidistant configuration minimizes $E_{\mathcal {F}}$ at any scale if $e^\rho$ minimizes $E_{\mathcal {F}}$ in $\mathcal {A}_N^\rho$ for all $\rho >0$ and all $N\in 2\mathbb {N}$. Furthermore, we say that the equidistant configuration minimizes $E_{\mathcal {F}}$ at high density if there exists $\rho _0$ such that for all $N\in 2\mathbb {N}$ and all $\rho >\rho _0$, $e^\rho$ is a minimizer of $E_{\mathcal {F}}$ in $\mathcal {A}_N^\rho$.

In [36], Ventevogel proved the optimality of $e^\rho$ for identical particles when, for any $\alpha ,\beta \in \{1,2\}$, $f_{\alpha \beta }=f$ is a convex function. The following theorem generalizes this result for two kinds of alternating species and three kinds of interactions, also including some classes of nonconvex functions:

### Theorem 2.2

(Sufficient condition) Suppose that $f_{\alpha \beta }(x)=\Phi _{\alpha \beta }^+(|x|)-\Phi _{\alpha \beta }^-(|x|)$ for convex and strongly tempered functions $\Phi _{\alpha \beta }^\pm : [0,\infty ) \rightarrow {\mathbb {R}}$ for any $\alpha , \beta \in \{1,2\}$ in the sense that there exists $r_0,C,\eta >0$ such that

$$\begin{aligned} |\Phi _{\alpha \beta }^\pm (r)| < C r^{-1-\eta } \quad \text { for } r >r_0. \end{aligned}$$

Moreover, suppose that the function *F* is convex on $(0,+\infty )$, where

2.1 $$\begin{aligned} F(r) :=2\Phi _{12}^+(r)- \sum _{k=1}^\infty \big ( \Phi _{12}^-((2k-1)r) + \Phi _{22}^-(2kr) + \Phi _{11}^-(2kr) \big ). \end{aligned}$$

Then the equidistant configuration is the unique minimizer of $E_{\mathcal {F}}$ at any scale.

**Fig. 2:**
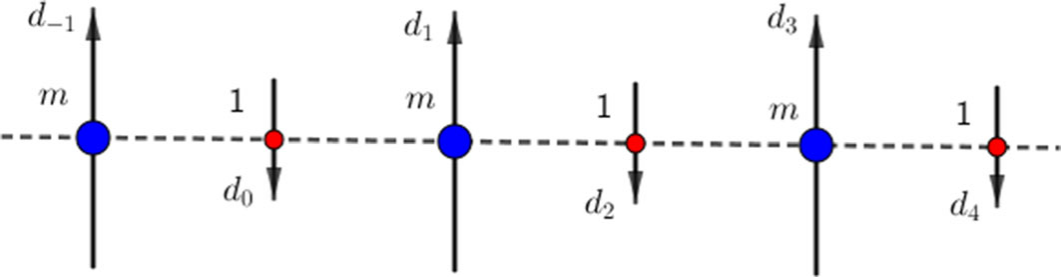
System of alternate oriented dipoles of magnitude 1 and *m*

We now give a direct application of Theorem 2.2 for systems with alternating charges $1,-m$ and power-law interaction potential, in the integrable case. We can think about two kinds of individuals with “mass” 1 and *m* interacting via the potentials $x\mapsto \pm |x|^{-p}$. The following result shows that once *p* is fixed, there exists an interval of *m* containing $m=1$ such that the equidistributed configuration is the only minimizer of the energy at any scale. It gives (non-optimal) bounds on *m* such that this equilibrium is achieved. Another physical motivation are chains of antiparallel dipoles which are common e.g. in the self-assembly of magnetic nanoparticles [33, Fig. 1] and classical models of spin chains [5, Sect. 3] where this regular structure reaches the equilibrium when there is no anisotropy field. Let us consider the following toy model of a chain of dipoles $d_{n}$, located at position $(x_{n},0,0) \in {\mathbb {R}}^3$ for $n \in \mathbb {Z}$. The dipoles are aligned in direction of the $x_2$ axis with alternating orientation and with magnitude given by $|d_{2k}| = 1$ and $|d_{2k+1}|=m$ (see Fig. 2). The interaction potentials, up to a positive constant, are then given by $f_{12}(x)=-m |x|^{-3}$, $f_{11}(x)=|x|^{-3}$, $f_{22}(x)= m^2 |x|^{-3}$. Hence, the following result gives a condition on *m* and *p* such that the equidistant configuration is the only maximum for this system at any scale.

### Corollary 2.3

(Crystallization for the inverse power-law: non-neutral case) Let $f_{12}(x)=m |x|^{-p}$, $f_{11}(x)=-|x|^{-p}$ and $f_{22}(x)=-m^2 |x|^{-p}$, for $p>p_1$ where $p_1\approx 1.46498>1$ is the unique solution of $\zeta (p_1)=2^{p_1}$, where $\zeta (s):=\sum _{n>0} n^{-s}$ is Riemann’s zeta function, and let *m* be such that

2.2 $$\begin{aligned} m_p< m < \frac{1}{m_p}, \quad m_p:=\frac{2^p -\sqrt{4^p - \zeta (p)^2}}{\zeta (p)}. \end{aligned}$$

Then the equidistant configuration is the unique minimizer of $E_{\mathcal {F}}$ at any scale.

We also note that if *m* is sufficiently large (depending on any fixed *p*, *N*) then the equidistributed configuration cannot be a minimizer of $E_{\mathcal {F}}$, the dominant interaction being $f_{22}(x)=-m^2 |x|^{-p}$, which forces the particles to be close to each other.

As a consequence of Theorem 2.2, the Riesz potentials $f_{11}(x)= f_{22}(x) = - f_{12}(x) = |x|^{-p}$ are minimized by the equidistant configuration for any $p>1$. The next result improves this result to the case $p \ge p_0$ where $p_0 \approx 0.655$ is the unique solution of

2.3 $$\begin{aligned} \zeta (1+p_0)+1=2^{1+p_0} \quad \text { in }(0,\infty ). \end{aligned}$$

In particular, this includes the case of the three-dimensional Coulomb potential.

### Theorem 2.4

(Riesz potentials) Let

$$\begin{aligned} f_{12}(x)= - f_{11}(x)= - f_{22}(x) = \frac{1}{|x|^p} \quad \text {for } p\ge p_0, \end{aligned}$$

where $p_0$ is the unique solution of (2.3). Then the equidistant configuration is the unique minimizer of $E_{\mathcal {F}}$ at any scale.

### Remark 2.5

(*Non-optimality at any scale for different exponents*) If we consider the anisotropic system where

$$\begin{aligned} f_{12}(x)=\frac{1}{|x|^p},\quad \text {and}\quad f_{11}(x)=f_{22}(x)=-\frac{1}{|x|^q},\quad p\ne q, \end{aligned}$$

the crystallization on $e^\rho$ does not hold at any scale, contrary to what happens if $p=q$ as stated in Theorem 2.4. Indeed, if $p>q$ (resp. $p<q$), the fact that $|x|^{-p}=o(|x|^{-q})$ as $|x|\rightarrow \infty$ (resp. as $|x|\rightarrow 0$) implies that there exists a density $\rho _0$ (resp. $\rho _1$) such that for all $\rho <\rho _0$ (resp. $\rho >\rho _1$), $e^\rho$ is not a minimizer of $E_{\mathcal {F}}$ in $\mathcal {A}_N^\rho$ since the main term of the energy is then attractive for this range of densities.

We notice that the alternation of species is the only case where the minimizer does not have two points at the same location. Indeed, if two points of the same kind are adjacent, it is sufficient to merge them in order to get an energy equal to $-\infty$. In order to improve Theorem 2.2 for non-summable potentials, the use of the homogeneity is a key point. In particular, Theorem 2.4 shows the maximality of the alternate equidistant configuration (with charges $\pm 1$), at any scale, for the standard Coulomb energy. Notice also that Theorem 2.4 is reminiscent of a result by Hubbard [22] and Pokrovsky-Uimin [28] on lattice spin systems (see also [3]) but where the setting is slightly different: electrons are interacting through a Coulomb (or Riesz) interaction but a certain number of them are prescribed in a period and their presence at lattice sites are discussed. In a sense, there is no competition of two energies with totally opposite behavior in the latter as it is the case in our model.

### Remark 2.6

(*Limit of our method and universal optimality*) We believe (supported by numerical experiments) that the equidistant configuration should be optimal for any $p > 0$ (including even the logarithmic potential). However, the constraint on the parameter $p_0$ is related to out method. We also believe that a new type of universal optimality—in the sense of Cohn and Kumar [14]—holds for such alternate systems, which leads to the following conjecture.

### Conjecture 2.7

(Universal optimality for alternating chains) For any *f* such that $f(r)=F(r^2)$ and *F* is a completely monotone function (i.e. the Laplace transform of a nonnegative Borel measure), $e^\rho$ is the unique minimizer of $E_{\mathcal {F}}$, where ${\mathcal {F}}:=(-f,-f,f)$, at any scale.

Theorem 2.4 already proves this fact for a subclass of completely monotone potentials. However, using the linear programming bounds method as in [14, 15] seems to be challenging—even in dimension 1—for this type of alternate chain of particles. Furthermore, we expect Conjecture 2.7 to hold in higher dimensions $d\in \{2,4,8,24\}$ for the respective best packing and a more general class of functions.

In order to explore a physically relevant anisotropic case, we now assume that the particles of the same type interact through the purely repulsive potential $f_{11}=f_{22}$ while the opposite type of particles are interacting through the attractive-repulsive one-well potential $f_{12}$, respectively given by

2.4 $$\begin{aligned} f_{11}(x)=f_{22}(x) =\frac{1}{|x|^p}+\frac{1}{|x|^q}, \quad f_{12}(x) = \frac{1}{|x|^p}-\frac{1}{|x|^q},\quad p>q>1. \end{aligned}$$

This model is known as a good model for ionic interactions, because it takes into consideration the Pauli exclusion principle by being repulsive at the origin, which avoids the collapsing of the ground state. We therefore show, combining Theorems 2.2 and 2.4, that the equidistant configuration is the unique minimizer of $E_{\mathcal {F}}$ among configurations of sufficiently high density.

### Theorem 2.8

(Optimality at high density in an anisotropic case) Let $f_{ij}$ be defined by (2.4). Then there exists $\rho _0$ such that for all $\rho >\rho _0$ and all $N\in \mathbb {N}$, $e^{\rho }$ is the unique minimizer of $E_{\mathcal {F}}$ in ${\mathcal A}_N^\rho$. Furthermore, if $\rho <\rho _0$, then $e^\rho$ is not a minimizer of $E_{\mathcal {F}}$ in ${\mathcal A}_N^\rho$.

Notice that we have derived an analogous result in [7] in any dimension in the case of (orthorhombic) Bravais lattices, showing the minimality of the rock-salt structure. Furthermore, this type of high density result is usually a consequence of the universal optimality of a structure, as it is done for instance in [6, 7] and we think that Conjecture 2.7 could lead to a general high density result including a Coulombian tail $q=1$ in the same spirit as the two-dimensional result [6, Thm. 1.1].

Finally, we recall a necessary condition for the optimality of the equidistant configuration at high density, which has been derived by Ventevogel and Nijboer’s result in a related setting of identical particles [37], based on the notion of strongly tempered potentials (cf. also Süto [34]).

### Proposition 2.9

(Necessary condition for high density crystallization) Suppose that the functions $f_{\alpha \beta } \in C^2({\mathbb {R}})\cap L^1({\mathbb {R}})$ are mirror symmetric and strongly tempered for any $\alpha ,\beta \in \{1,2\}$, in the sense that there exists $r_0,C,\eta >0$ such that

$$\begin{aligned} |f_{\alpha \beta } (x)| < C|x|^{-1-\eta } \quad \text { for any }|x|>r_0. \end{aligned}$$

If the equidistant configuration is a minimizer of $E_{\mathcal {F}}$ at high density, then

$$\begin{aligned} \widehat{f_{12}}(k)+\frac{1}{2}\big (\widehat{f_{11}}(k)+\widehat{f_{22}}(k) \big )\ge 0&\qquad \qquad \text {for all } k\in {\mathbb {R}}, \end{aligned}$$

where $\widehat{f}(k)=\frac{1}{\sqrt{2\pi }}\int _{\mathbb {R}}f(x)\cos (kx)dx$ is the Fourier transform of *f*.

The positivity of the Fourier transform of interaction potential seems to play a crucial role in optimal point configurations theory (see e.g. [14, 15, 34]). For instance, Nikos et al. have shown in [26]—for a generalized exponential model with one type of particles–an equivalence between the emergence of clusters and the existence of negative Fourier components of the interaction potential. Proposition 2.9 suggests that results in the same direction could be obtained for two-components systems.

## Proofs

In the following proofs, we use the notation $\ell :=\rho ^{-1}$.

### Proof of Theorem 2.2

In this proof, for convenience, we write $\Phi _{\alpha \beta }^\pm (x)$ instead of $\Phi _{\alpha \beta }^\pm (|x|)$. In view of the assumptions of the theorem, for any $X\in {\mathcal A}_N^\rho$ we have,

$$\begin{aligned} E_{\mathcal {F}}(X)&=\frac{2}{N}\sum _{n=1}^N \sum _{k=1}^\infty \Phi _{12}^+(x_{n+2k-1}-x_n) + \frac{2}{N}\sum _{j=1}^{N/2}\sum _{k=1}^\infty \Phi _{22}^+(x_{2j+2k}-x_{2j})\\&\quad +\frac{2}{N}\sum _{j=1}^{N/2}\sum _{k=1}^\infty \Phi _{11}^+(x_{2j-1+2k}-x_{2j-1})-\frac{2}{N}\sum _{n=1}^N \sum _{k=1}^\infty \Phi _{12}^-(x_{n+2k-1}-x_n)\\&\quad -\frac{2}{N}\sum _{j=1}^{N/2} \sum _{k=1}^\infty \Phi _{22}^-(x_{2j+2k}-x_{2j})-\frac{2}{N}\sum _{j=1}^{N/2} \sum _{k=1}^\infty \Phi _{11}^-(x_{2j-1+2k}-x_{2j-1} )\\&=: S_1+S_2+S_3-S_4-S_5-S_6. \end{aligned}$$

We estimate the six expressions using convexity of the functions and periodicity. By convexity of $\Phi _{12}^+$ and with the notation $d_n := x_{n+1}-x_n$, we have by Jensen’s inequality

$$\begin{aligned} S_1&\ge \frac{2}{N}\sum _{n=1}^N \Phi _{12}^+(d_n)+2\sum _{k=2}^\infty \Phi _{12}^+\big ((2k-1)\ell \big ). \end{aligned}$$

By convexity of $\Phi _{22}^+$, we furthermore obtain, using Jensen’s inequality,

$$\begin{aligned} S_2&\ge \sum _{k=1}^\infty \Phi _{22}^+\Big ( \frac{2}{N}\sum _{j=1}^{N/2} x_{2j+2k}-x_{2j}\Big )=\sum _{k=1}^\infty \Phi _{22}^+\big (2k\ell \big ), \end{aligned}$$

the same holds for $S_3$ replacing $\Phi _{22}^+$ by $\Phi _{11}^+$. For the terms $S_4$, $S_5$, $S_6$ with a negative sign, we decompose into nearest-neighbours distances. By convexity of $\Phi _{12}^-$ we get, again by Jensen’s inequality

$$\begin{aligned} S_4&=\frac{2}{N}\sum _{n=1}^N \sum _{k=1}^\infty \Phi _{12}^- \Big ( \sum _{m=1}^{2k-1} d_{n+m-1} \Big ) \le \frac{ 2}{N}\sum _{n=1}^N \sum _{k=1}^\infty \Phi _{12}^-((2k-1) d_n). \end{aligned}$$

Similarly, by convexity of $\Phi _{22}^-$ and $\Phi _{11}^-$, we obtain

$$\begin{aligned} S_5&=\frac{2}{N}\sum _{j=1}^{N/2} \sum _{k=1}^\infty \Phi _{22}^-\Big ( \sum _{m=1}^{2k} d_{2j+m-1} \Big ) \le \frac{2}{N}\sum _{j=1}^{N/2} \sum _{k=1}^\infty \frac{1}{2k}\sum _{m=1}^{2k} \Phi _{22}^-(2k d_{2j+m-1}) \\&\le \frac{1}{N} \sum _{n=1}^N \sum _{k=1}^\infty \Phi _{22}^-(2k d_n), \\ S_6&=\frac{2}{N}\sum _{j=1}^{N/2} \sum _{k=1}^\infty \Phi _{11}^- \Big ( \sum _{m=1}^{2k} d_{2j+m-2} \Big ) \le \frac{1}{N} \sum _{n=1}^N \sum _{k=1}^\infty \Phi _{11}^-(2k d_n). \end{aligned}$$

Combining all these inequalities and since *F* given by (2.1) is convex, we have, by Jensen’s inequality,

$$\begin{aligned} E_{\mathcal {F}}(X)&\ge \ 2\sum _{k=1}^\infty \Phi _{12}^+\left( (2k+1)\ell \right) +\sum _{k=1}^\infty \Phi _{22}^+\left( 2k\ell \right) +\sum _{k=1}^\infty \Phi _{11}^+\left( 2k\ell \right) +\frac{1}{N}\sum _{n=1}^N F(d_n) \\&\ge 2\sum _{k=1}^\infty \Phi _{12}^+\left( (2k+1)\ell \right) +\sum _{k=1}^\infty \Phi _{22}^+\left( 2k\ell \right) +\sum _{k=1}^\infty \Phi _{11}^+\left( 2k \ell \right) +F\left( \ell \right) = E_{\mathcal {F}}(e^\rho ), \end{aligned}$$

with equality if and only if $X=e^\rho$.

### Proof of Theorem 2.4

The main idea is to compare the interaction on distances $|x_i - x_j|$ where $i-j$ is even with interactions of distances where $i-j$ is odd: We use the convex combination

3.1 $$\begin{aligned} x_{n+2k}-x_n = \frac{(2k-j)}{2k} \frac{2k(x_{n+2k}-x_{n+j})}{2k-j} + \frac{j}{2k}\frac{2k(x_{n+j}-x_{n})}{j}, \end{aligned}$$

which holds for all $1\le j \le k$. We set $f(x) := |x|^{-p}$ and use again the notation $d_n := x_{n+1}-x_n$. Inserting (3.1) for $j=1$ into *f* and exploiting convexity, we get

$$\begin{aligned} f(x_{n+2k}-x_n) \le \frac{2k-1}{2k}f\left( \frac{2k(x_{n+2k}-x_{n+1})}{2k-1}\right) + \frac{1}{2k}f(2kd_{n}). \end{aligned}$$

Since *f* is homogeneous of degree $-p$, the last line implies

3.2 $$\begin{aligned} f(x_{n+2k}-x_n) \le \Big (\frac{2k-1}{2k}\Big )^{1+p}f\left( x_{n+2k}-x_{n+1}\right) + \Big (\frac{1}{2k}\Big )^{1+p}f(d_n). \end{aligned}$$

Averaging (3.2) over *n* and using periodicity of *X*, we get

$$\begin{aligned} \frac{1}{N} \sum _{n=1}^N f(x_{n+2k}-x_n) \le \frac{1}{N} \sum _{n=1}^N \Big (\Big (\frac{2k-1}{2k}\Big )^{1+p}f(x_{n+2k-1}-x_{n}) + \Big (\frac{1}{2k}\Big )^{1+p}f(d_n)\Big ), \end{aligned}$$

i.e. a bound on the interaction on even distances in terms of the interaction on odd distances. Inserting this estimate into the energy $E_{\mathcal {F}}$ yields the lower bound

$$\begin{aligned} E_{\mathcal {F}}(X)\ge \frac{2}{N}\sum _{n=1}^N\sum _{k=1}^{\infty } a_kf(x_{n+2k-1}-x_n), \end{aligned}$$

where the coefficients $a_k$ are given by

$$\begin{aligned} a_k:= {\left\{ \begin{array}{ll} 1-\frac{1}{2^{1+p}}-\sum _{j=1}^\infty \left( \frac{1}{2j}\right) ^{1+p} =1- 2^{-(1+p)}(\zeta (1+p)+1) &{} \text {for }k=1,\\ 1-\left( \frac{2k-1}{2k}\right) ^{1+p} &{} \text {otherwise}. \end{array}\right. } \end{aligned}$$

Since $a_1:p\mapsto 1- 2^{-(1+p)}(\zeta (1+p)+1)$ is an increasing function on $(0,\infty )$, $p_0$ is unique and $p\ge p_0$ implies that $a_k \ge 0$ for all $k \ge 1$. Applying Jensen’s inequality and inserting $\frac{1}{N}\sum _{n=1}^{N} (x_{n+2k-1}-x_n) = (2k-1)\ell$ yields the lower bound

$$\begin{aligned} E_{\mathcal {F}}(X) \ge 2\sum _{k=1}^{\infty }a_kf\left( (2k-1)\ell \right) = E_{\mathcal {F}}(e^\rho ), \end{aligned}$$

which is strict unless $X=e^\rho$, corresponding to equality in Jensen’s inequality.

### Proof of Corollary 2.3

We want to apply Theorem 2.2. We have

$$\begin{aligned} F(r) = \frac{2m}{r^{p}}-\frac{(m^2 +1)\zeta (p)}{2^p r^{p}},&F''(r) = \Big (-\frac{\zeta (p)}{2^p}m^2 +2m -\frac{\zeta (p)}{2^p} \Big )\frac{p(p+1)}{r^{p+2}}. \end{aligned}$$

The discriminant of the polynomial $P_p(m):=-\frac{\zeta (p)}{2^p}m^2 +2m -\frac{\zeta (p)}{2^p}$ is $\Delta =4\big ( 1-\frac{\zeta (p)^2}{2^{2p}} \big )$, is positive if and only if $\zeta (p)<2^p$, since $p\mapsto 2^p -\zeta (p)$ is increasing on $(1,+\infty )$. Then, $P_p(m)>0$ if and only if *m* satisfies (2.2), and the proof is completed.

### Proof of Theorem 2.8

We first remark that, for any $\rho >0$, any $N\in \mathbb {N}$ and any $X\in \mathcal {A}_N^\rho$, we have

$$\begin{aligned} E_{\mathcal {F}}(X)&=E_{{\mathcal {F}}_p}(X)+E_{{\mathcal {F}}_q}(X),\quad {\mathcal {F}}_p:=\left( \frac{1}{|x|^p},\frac{1}{|x|^p}, \frac{1}{|x|^p} \right) ,\quad \\ {\mathcal {F}}_q&:=\left( \frac{1}{|x|^q},\frac{1}{|x|^q}, -\frac{1}{|x|^q} \right) . \end{aligned}$$

By the homogeneity of $f_{ij}$ and for the rescaled configuration $X^1=\rho ^{-1}X\in \mathcal {A}_N^1$, we have $E_{\mathcal {F}}(X)=\rho ^p E_{{\mathcal {F}}_p}(X^1)+\rho ^{q}E_{{\mathcal {F}}_q}(X^1)$. By Theorem 2.4 (resp. Theorem 2.2), we know that $E_{{\mathcal {F}}_q}(X^1)\le E_{{\mathcal {F}}_q}(e^1)$ (resp. $E_{{\mathcal {F}}_p}(X^1)\ge E_{{\mathcal {F}}_p}(e^1)$) with equality if and only if $X^1=e^1$. Hence, $E_{\mathcal {F}}(X)\ge E_{\mathcal {F}}(e^\rho )$ for all $X\in \mathcal {A}_N^\rho$ if and only if

3.3 $$\begin{aligned} \rho > \rho _0 \ := \ {\mathop {\mathop {\mathrm {sup}}\limits _{X^{1}\in {\mathcal {A}}_N^{1}}} \limits _{X_1\ne e^1}}\left( \frac{E_{{\mathcal {F}}_q}(e^1)-E_{{\mathcal {F}}_q}(X^1)}{E_{{\mathcal {F}}_p}(X^1)-E_{{\mathcal {F}}_p}(e^1)}\right) ^{\frac{1}{p-q}}. \end{aligned}$$

It remains to show that $\rho _0 < +\infty$. Let $\Vert \cdot \Vert$ be the Euclidean norm on one period, with total length *N*, of any configuration belonging to ${\mathcal A}_N^1$. We notice that $X^1\mapsto E_{{\mathcal {F}}_q}(e^1)-E_{{\mathcal {F}}_q}(X^1)$ and $X^1\mapsto E_{{\mathcal {F}}_p}(X^1)-E_{{\mathcal {F}}_p}(e^1)$ are both continuous in $\mathcal {A}_N^1$ with respect to $\Vert \cdot \Vert$ and vanish if and only if $X^1=e^1$. We also notice that the fact that $|x|^{-q}=o(|x|^{-p})$ as $x\rightarrow 0$ implies that any coalescence of two points of $X^1$ gives a zero energy contribution since the denominator tends to infinity faster than the numerator. Therefore, the above quotient is continuous on ${\mathcal A}_N^1\backslash \{e^1\}$.

By strict minimality (resp. maximality) of $e^1$ for $E_{{\mathcal {F}}_p}$ (resp. $E_{{\mathcal {F}}_q}$) and the Taylor expansion of these energies in $\mathcal {A}_N^1$ close to $e^1$ combined with the continuity of the energies and the fact that this strict minimality still holds in the limit $N\rightarrow \infty$, it follows that there exists a sufficiently small $\delta >0$ and a constant $C_\delta >0$ such that the right hand side of (3.3) is bounded by $C_\delta$ for all $X^1$ with $\Vert X^1-e^1\Vert <\delta$.

We now show that the above quotient is bounded above uniformly in *N* when $\Vert X^1-e^1\Vert \ge \delta$, $X^1\in {\mathcal A}_N^1$. Since we have already studied the behaviour of the above quotient as $X^1$ is sufficiently close to $e^1$, we only need consider the quotient $-E_{{\mathcal {F}}_q}/E_{{\mathcal {F}}_p}>0$, since $E_{{\mathcal {F}}_q}\le E_{{\mathcal {F}}_q}<0$ and $E_{{\mathcal {F}}_p}>0$ on ${\mathcal A}_N^1$, and to show that there exists $C>0$ such that

$$\begin{aligned} E_{p,q}(X^1):=C E_{{\mathcal {F}}_p}(X^1) + E_{{\mathcal {F}}_q}(X^1)\ge 0, \qquad \quad \forall N, \quad \forall X^1 = \{x_i\}_{i\in \mathbb {Z}} \in {\mathcal A}_N^1. \end{aligned}$$

Using that $r^{-p}\ge r^{-q}$ if and only if $r\le 1$, we get

$$\begin{aligned} E_{p,q}(X^1)&= \ \frac{1}{2N}\sum _{i=1}^N \sum _{x_j \in X^1 \backslash \{x_i\}} \left\{ \frac{C}{|x_i-x_j|^p} + \frac{(-1)^{i-j}}{|x_i-x_j|^q} \right\} \\&\ge \frac{1}{2N}\sum _{i=1}^N \sum _{\begin{array}{c} x_j \in X^1\backslash \{x_i\}:i-j\in 2\mathbb {Z}+1 \\ |x_i-x_j| \le 1 \end{array}} \frac{C-1}{|x_i-x_j|^p} - \frac{1}{2N}\sum _{i=1}^N \sum _{\begin{array}{c} x_j \in X^1: i-j \in 2\mathbb {Z}+1 \\ |x_i-x_j| > 1 \end{array}} \frac{1}{|x_i-x_j|^q} \\&\ge \ \frac{1}{2N}\left( C-1 - C_2\right) . \end{aligned}$$

For the last estimate, we have used that the first sum contains at least one term by the pigeonhole principle and we have assumed that $C\ge 1$. For the estimate of the second term, we have used that the double sum is uniformly bounded because

$$\begin{aligned} \sum _{i=1}^N \sum _{\begin{array}{c} x_j \in X^1: i-j \in 2\mathbb {Z}+1 \\ |x_i-x_j|> 1 \end{array}} \frac{1}{|x_i-x_j|^q}&\le \ C_1 \iint _{(x,y)\in {\mathbb {R}}^2: |x-y|>1} \frac{dxdy}{|x-y|^q}< C_2 < \infty , \end{aligned}$$

since $q>1$, where the constants $C_1,C_2$ only depend on *p*, *q*. For $C := 1+C_2$ we get $E_{p,q}(X^1)\ge 0$, which completes the proof.

### Proof of Proposition 2.9

We adapt the proof of [37, Sect. 2] to our case. By assumption, we have, for any *N* and any $0<\ell <\ell _0$, $E_{\mathcal {F}}(X)\ge E_{\mathcal {F}}(e^\rho )$ for any $X = (x_i)_{i \in \mathbb {Z}} \in {\mathcal A}_N^\rho$. We choose in particular $x_n :=y_n+n\ell -\epsilon$ with $y_n=\epsilon \cos \left( \frac{2\pi m n}{N} \right)$ for some small $\epsilon >0$ such that $\epsilon <\ell /2$ and for some $m\in \mathbb {Z}$. With this choice, we have $x_0 = 0$ and $x_{i+N}-x_i=N\ell$ and $x_{i+1}-x_i>0$ for any $i\in \mathbb {Z}$, and hence $X\in {\mathcal A}_N^\rho$. Using Taylor expansion, by minimality of the equidistant configuration we hence get, for $\ell$ sufficiently small,

$$\begin{aligned} \sum _{n=1}^N\sum _{j \in \mathbb {Z}}^\infty \left| y_j-y_n \right| ^2 f_{\epsilon _j\epsilon _n}''((j-n)\ell ) \ge 0. \end{aligned}$$

Hence there is $\ell _1>0$ such that for any $\ell \le \ell _1\le \ell _0$ and $g(x):=\frac{1}{2}(f_{11}(x)+f_{22}(x))$,

3.4 $$\begin{aligned} \sum _{j \in \mathbb {Z}} \left( 1-\cos \left( (2j-1)q \right) \right) f_{12}''((2j-1)\ell )+\sum _{j\in \mathbb {Z}}\left( 1-\cos \left( 2q j\right) \right) g''(2j\ell )\ge 0, \end{aligned}$$

where $q := \frac{2\pi m}{N}$. Since (3.4) holds independently of *N*, by an approximation argument we also have for any $x\in {\mathbb {R}}$ and any $0<\ell \le \ell _1\le \ell _0$,

3.5 $$\begin{aligned} \sum _{j \in \mathbb {Z}} (1-\cos (2j x-x)f_{12}''((2j-1)\ell )+\sum _{j\in \mathbb {Z}} (1-\cos (2j x)) g''(2j\ell )\ge 0. \end{aligned}$$

Thus, multiplying (3.5) by $\ell$, taking $x=\ell k$ and dividing by $k^2$, we get

$$\begin{aligned} 0&\le \lim _{\ell \rightarrow 0} \Big (\ell \sum _{j \in \mathbb {Z}} \frac{(1-\cos ( (2j \ell -\ell )k)}{k^2}f_{12}''((2j-1)\ell )+\ell \sum _{j \in \mathbb {Z}} \frac{(1-\cos (2j k\ell ))}{k^2}g''(2j\ell )\Big )\\&=\int _{ {\mathbb {R}}} \frac{1-\cos ((2y-1)k)}{k^2}f_{12}''(2y-1)dy+\int _{ {\mathbb {R}}} \frac{1-\cos (2yk)}{k^2}g''(2y)dy \\&= \widehat{f}_{12}(k) + \widehat{g}(k) \quad \text {for all}\;k \in {\mathbb {R}}\backslash \{ 0 \}. \end{aligned}$$
